# Prevalence of Varicose Veins Among Nurses in Different Departments in Jazan Public Hospitals, Saudi Arabia: A Cross-Sectional Study

**DOI:** 10.7759/cureus.24462

**Published:** 2022-04-25

**Authors:** Suhaila A Ali, Waad K Najmi, Fatimah M Hakami, Alzahra A Almubarak, Raimaa A Alhassan, Shahad H Maafa, Maryam A Al-Amer, Ibrahim M Dighriri

**Affiliations:** 1 Family and Community Medicine Department, Jazan University, Jazan, SAU; 2 Medicine, College of Medicine, Jazan University, Jazan, SAU; 3 PharmD, Jazan University, King Abdulaziz Specialist Hospital, Taif, SAU

**Keywords:** jazan, risk factor, lower limbs, nurses, varicose veins

## Abstract

Varicose veins (VV) in the lower limbs are the most prevalent vascular condition in humans. They can cause significant signs and symptoms and, in extreme cases, death. This study aims to investigate the prevalence and identify the risk factors for varicose veins among nurses working in multiple departments at Jazan King Fahd Central Hospital and Prince Muhammad bin Nasser Hospital. A cross-sectional study was conducted by sending a questionnaire to female and male nurses in these hospitals. This study included 482 nurses, 415 (86.1%) of whom were female and 67 (13.9%) male. The prevalence of varicose veins among the nurses of both hospitals was 76 (15.8%), compared with 406 (84.2%) not diagnosed with varicose veins. The prevalence of varicose veins was 67 (88.2%) in female nurses, compared with 9 (11.8%) in male nurses. The risk factors associated with varicose veins were ethnicity (*p *= 0.007), carrying heavy items (*p *= 0.001), lack of exercise (*p* = 0.031), family history (*p *= 0.001), use of hormonal therapy (*p *= 0.001), use of contraceptive pills (*p *= 0.0035), type of delivery (*p *= 0.002), number of children (*p *= 0.004), and hours sitting per shift (*p = *0.002). The comorbidities associated with varicose veins were deep vein thrombosis (*p *= 0.001), hypertension (*p *= 0.002), chronic constipation (*p *= 0.006), diabetes (*p *= 0.001), kidney disease (*p *= 0.001), rheumatoid arthritis (*p *= 0.001), coronary artery disease, and severe occupational injury to the lower extremities (*p *= 0.001). Nurses are responsible for most of the health system's services. Increasing the number of nursing workers for patient care, encouraging physical exercise, and lowering the pension age appear to be required to avoid the occurrence and development of varicose veins among nurses.

## Introduction

Varicose veins (VV) in the lower limbs are the most prevalent vascular condition in humans, causing significant signs and symptoms and, in extreme cases, death [1,2]. VV is indicated by a 3 to 4 mm dilatation of the subcutaneous veins [3]. There are several types of vein diseases, including reticular, telangiectasia, and trunk veins [4]. VV may cause pain in the afflicted limbs and aesthetic difficulties, which often require surgery or other intensive treatments and deplete the healthcare budget [5]. The vein is constructed of tiny valves that prevent blood flow from reversing. The malfunction of these valves allows the reversal of blood flow to reverse and accumulate in the veins, increasing their prominence and enlargement [6,7].

Numerous factors may contribute to the valves' weakening and failure, including genetics, venous thrombosis, and the loss of the elastic tissue of the venous wall [8]. In the lower limbs, varicose veins symptoms may be localized to the afflicted region or can be widespread, affecting the whole lower leg. Localized symptoms such as pain, burning, and itching are prevalent, but leg pains, heaviness, weariness, and edema are also typical. The symptoms are typically intense during prolonged periods of standing and subside when patients sit and raise their legs [9]. Complications from varicose veins are rare. However, clots in the deep blood veins of the legs can sometimes expand. The afflicted leg is painful and swollen in such circumstances, and any chronic leg discomfort or swelling requires medical treatment. Because it may indicate a blood clot, it is medically referred to as thrombophlebitis. Additionally, the skin around varicose veins may develop painful ulcers, particularly around the ankles. Usually, a discolored area on the skin extends prior to the formation of an ulcer. Veins that are extremely near to the surface of the skin may rupture, often resulting in relatively minimal bleeding [10].

The examination of varicose veins should be conducted while standing in good light to determine the degree and size of the varicose veins and the presence of other venous disorders. The presence or absence of venous hypertension-related skin injuries is the most critical medical concern [11]. Continuous-wave Doppler testing is an excellent tool for identifying saphenofemoral and saphenopopliteal junction reflux in varicose vein patients. In comparison with a duplex ultrasound exam, continuous-wave Doppler testing should be suggested to manage varicose veins due to its shorter evaluation time and ease of use in the clinical setting [12]. If the patient exhibits no symptoms or pain, therapy may be unnecessary; however, if the patient shows signs and symptoms, treatment may be essential to alleviate aches and pains and address concerns such as leg ulcers, skin discoloration, or swelling. Several therapies include surgery, endovascular therapy, and compression therapy [12].

Nurses are critical components of healthcare systems, and lower leg varicose veins may result in performance instabilities and increased economic expenditures by people and society to remedy the imposed damage. The issue is essential, as it may imperil work production, health in old age, various underlying thrombotic events, and other grave consequences [13]. This study aims to investigate the prevalence and identify the risk factors for varicose veins among nurses working in multiple departments at Jazan King Fahd Central Hospital and Prince Muhammad bin Nasser Hospital.

## Materials and methods

Study design and area

A cross-sectional study was conducted at King Fahd Central Hospital and Prince Muhammad bin Nasser Hospitals in Jazan province. Jazan (also called Gizan) is one of the 13 provinces of the Kingdom of Saudi Arabia (KSA). It is located in the southwest of the KSA and is highly populated with 1.5 million residents.

Identification of study participants

The sample size was calculated using the Raosoft online calculator, and it was estimated to be 482 nurses. The sampling was divided into males and females and stratified according to different departments at King Fahd Central Hospital and Prince Muhammad bin Nasser Hospital.

Inclusion and exclusion criteria

All hired nurses of both genders working in administrative or clinical positions were included in the study. Pregnant nurses and nurses under the age of 18 years were excluded.

Data collection process

The demographic (age, gender, and ethnicity), behavioral (smoking daily or exercising), physical (height), and work-related variables (sitting and standing periods during work hours and handling heavy objects) sections of the questionnaire were all subjectively assessed by the participants. Health-related data (comorbidities, hormonal therapy/contraceptive use, number of gravida/parities, etc.) were also included in the questionnaire. We started collecting data after receiving approval from the Institutional Review Board (IRB) of the Jazan Health Ethics Committee; the approval number is 2197.

Data analysis

The data obtained were analyzed using the Statistical Package for Social Sciences (SPSS) software (IBM Corp., Armonk, NY). Before entering the data into SPSS, it was cleaned and described using descriptive statistics. To specify categorical variables such as gender and race, frequencies/percentages were employed. The mean and standard deviation explained quantitative variables such as weight, height, and birth rate. The chi-square or Fisher's exact test determines the associations between the outcomes and any categorical variables. A p-value of 0.05 was used for statistical significance.

## Results

This study included 482 nurses, of whom 415 (86.1%) were female and only 67 (13.9%) were male. Almost all participants were Arabian (374, 77.6%) with 106 (22%) Asian. Two hundred fifty-four (52.7%) were married, 217 (45%) were single, 9 (1.9%) were divorced, and 2 (0.4%) were widowed. More than half of the nurses worked at King Fahd Central Hospital (243, 50.4%), while 239 (49.6%) nurses were from Prince Muhammad bin Nasser Hospital. The prevalence of varicose veins among the nurses of both hospitals was 76 (15.8%), compared with 406 (84.2%) not diagnosed with varicose veins. Four hundred four (83.8%) were nonsmokers; only 78 (16.2%) of the participants were smokers. Almost half of all participants did not play sports (238, 49.4%), while 206, (42.7%) did four times or less weekly. The majority of participants, 71 (35.5%), had been working for one to four years, and 136 (28.2%) had been working for five to nine years. Many of the nurses, 152 (31.5%), worked five shifts weekly, and 105 (21.8%) worked three shifts weekly (Table 1).

**Table 1:** Demographic characteristics of nurses working at King Fahd Central Hospital and Prince Muhammad bin Nasser Hospital in Jazan.

Variable		Frequency (%)
Gender	Female	415 (86.1%)
	Male	67 (13.9%)
Ethnicity	Arabian	374 (77.6%)
	Asian	106 (22%)
	African	1 (0.2%)
	Hispanic	1(0.2%)
Social status	Married	254 (52.7%)
	Single	217 (45%)
	Widow	2 (0.4%)
	Divorced	9 (1.9%)
The hospital you are working in	Prince Muhammad bin Nasser Hospital	239 (49.6%)
	King Fahd Central Hospital	243 (50.4%)
Are you a smoker?	Yes	78 (16.2%)
	No	404 (83.8%)
Do you usually lift objects weighing 23 kg or more during work time	Yes	264 (51%)
	No	236 (49%)
How often do you play sports or perform physical exercise per week?	No	238 (49.4%)
	Four times or less	206 (42.7%)
	Five times or more	38 (7.9%)
How many years working in the field	Less than one year	37 (15.1%)
	1–4 years	71 (35.5%)
	5–9 years	136 (28.2%)
	Ten years or more	102 (21.2%)
How many shifts do you have per week?	One shift	21 (4.4%)
	Two shifts	49 (10.2%)
	Three shifts	105 (21.8%)
	Four shifts	72 (14.9%)
	Five shifts	152 (31.5%)
	Six shifts	83 (17.2%)
Have you ever been diagnosed with varicose veins?	Yes	76 (15.8%)
	No	406 (84.2%)

**Figure 1:**
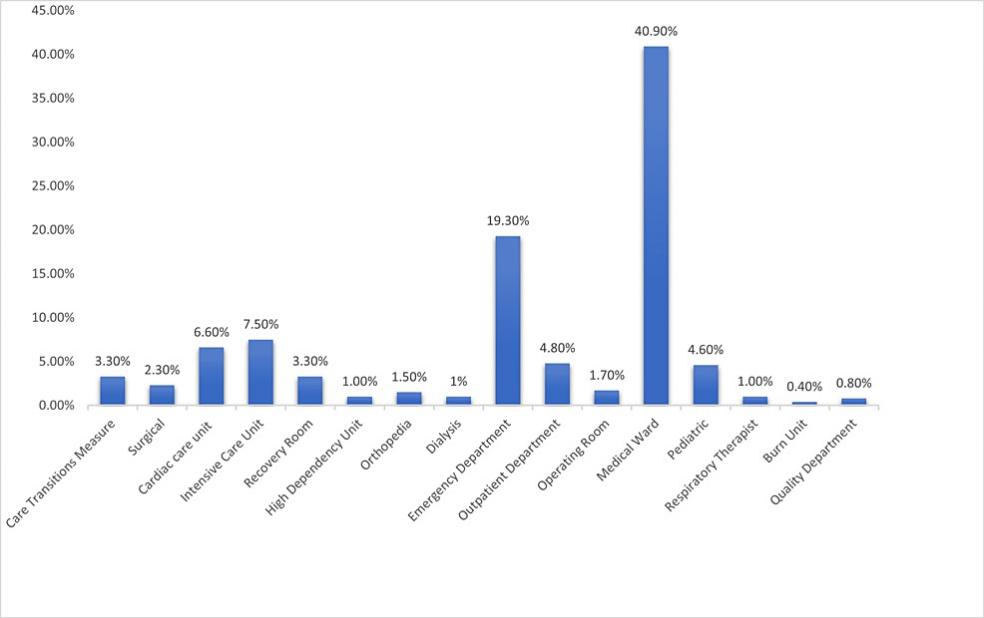
Departments of the nurses working at King Fahd Central Hospital and Prince Muhammad bin Nasser Hospital in Jazan.

Based on the work departments of the nurses, 40.90% of the participants worked in the medical ward, 19.30% in the emergency department, 7.50% in the intensive care unit, 6.60% in the cardiac care unit, and 25.7% in other departments (Figure 1).

The prevalence of varicose veins according to gender was 67 (88.2%) in female nurses, compared with 9 (11.8%) in male nurses. Fifty-one (67.1%) of the nurses diagnosed with varicose veins were married, compared with 24 (31.6%) single and 1 (1.3%) divorced. The number of positive responses for varicose veins working at King Fahd Central Hospital was 41 (53.9%). The prevalence of varicose veins was high in nurses carrying heavy items: 52 (68.4%). The incidence of varicose veins was higher for participants who did not play sports - 48 (63.2%) - compared with 35 (36.8%) for nurses who did. Only seven (9.2%) nurses diagnosed with varicose veins had been working for less than one year; in contrast, 27 (35.5%) participants diagnosed with varicose veins had been working for between five and nine years. Twenty-four (31.6%) nurses with varicose veins worked five shifts weekly, and 15 (19.7%) worked six shifts weekly. Thirty-two (42.1%) participants with varicose veins did not have children, compared with 44 (57.9%) who had children. Furthermore, 104 (25.6%) nurses were not diagnosed with varicose veins but were suspected of having varicose veins.

The risk factors associated with varicose veins were ethnicity (p=0.007), lifting heavy objects (p = 0.001), lack of exercise (p = 0.031), family history (p = 0.001), use of hormonal therapy (p = 0.001), use of contraceptive pills (p = 0.0035), type of delivery (p = 0.002), and number of children (p = 0.004). All these risk factors indicate statistically significant associations with varicose veins. The varicose vein predictors that were not statistically significant (p > 0.05) were gender, social status, place of work, number of shifts, years of work, and smoking. The comorbidities associated with varicose veins were deep vein thrombosis (p = 0.001), hypertension (p = 0.002), chronic constipation (p = 0.006), diabetes (p = 0.001), kidney disease (p = 0.001), rheumatoid arthritis (p = 0.001), coronary artery disease, and severe occupational injury to the lower extremities (p = 0.001). All these diseases had statistically significant associations with varicose veins (Table 2).

**Table 2:** Comparison of categorical variables of nurses with and without varicose veins.

Variable		With varicose veins frequency (%)	Without varicose veins frequency (%)	p-value
Gender	Female	67 (88.2%)	348 (85.7%)	0.572
	Male	9 (11.8%)	58 (14.3%)	
Ethnicity	Arabian	67 (88.2%)	307 (75.6%)	0.007
	Asian	8 (10.5%)	98 (24.1%)	
	African	1 (1.3%)	0 (0%)	
	Hispanic	0 (0%)	1 (0.2%)	
Social status	Married	51 (67.1%)	203 (50%)	0.053
	Single	24 (31.6%)	193 (47.5%)	
	Widow	0 (0%)	2 (0.5%)	
	Divorced	1 (1.3%)	8 (2%)	
The hospital you are working in	Prince Muhammad bin Nasser Hospital	35 (46.1%)	204 (50.2%)	0.502
	King Fahd Central Hospital	41 (53.9%)	202 (49.8%)	
Are you a smoker?	Yes	17 (22.4%)	61 (15.0%)	0.111
	No	59 (77.6%)	345 (85.0%)	
Do you usually lift objects weighing 23 kg or more during work time?	Yes	52 (68.4%)	194 (48.8%)	0.001
	No	24 (31.6%)	212 (52.2%)	
How often do you play sports or perform physical exercise per week?	No	48 (63.2%)	190 (46.8%)	0.031
	Four times or less	23 (30.2%)	183 (45.1%)	
	Five times or more	5 (6.6%)	33 (8.1%)	
How many years working in the field	Less than one year	7 (9.2%)	66 (16.3%)	0.257
	1–4 years	25 (32.9%)	146 (36%)	
	5–9 years	27 (35.5%)	109 (26.8%)	
	Ten years or more	17 (22.4%)	85 (20.9%)	
How many shifts do you have per week?	One shift	3 (3.9%)	18 (4.4%)	0.971
	Two shifts	8 (10.5%)	41 (10.1%)	
	Three shifts	14 (18.4%)	91 (22.4%)	
	Four shifts	12 (15.8%)	60 (14.8%)	
	Five shifts	24 (31.6%)	128 (31.5%)	
	Six shifts	15 (19.7%)	68 (16.7%)	
Do you suspect having varicose veins (permanently dilated, twisted, elongated superficial veins usually seen in the legs, maybe blue or dark purple, and often lumpy)?	Yes	56 (73.7%)	104 (25.6%)	0.001
	No	20 (26.3%)	302 (74.4%)	
Do you have a family history of varicose veins?	Yes	49 (64.5%)	88 (21.7%)	0.001
	No	27 (35.5%)	318 (78.3%)	
Do you have deep vein thrombosis (DVT)?	Yes	13 (17.1%)	18 (4.4%)	0.001
	No	63 (82.9%)	388 (95.6%)	
Do you have hypertension?	Yes	38 (50%)	129 (31.8%)	0.002
	No	38 (50%)	277 (68.2%)	
Do you have chronic constipation?	Yes	23 (30.3%)	68 (16.7%)	0.006
	No	53 (69.7%)	338 (83.3%)	
Do you have diabetes?	Yes	34 (44.7%)	105 (25.9%)	0.001
	No	42 (55.3%)	301 (74.1%)	
Do you have kidney disease?	Yes	17 (22.4%)	28 (6.9%)	0.001
	No	59 (77.6%)	378 (93.1%)	
Do you have rheumatoid arthritis?	Yes	20 (26.3%)	43 (10.6%)	0.001
	No	56 (73.7%)	363 (89.4%)	
Do you have coronary artery disease?	Yes	19 (25%)	24 (5.9%)	0.001
	No	57 (75%)	382 (94.1%)	
Do you have a severe occupational injury to the lower extremities?	Yes	22 (28.9%)	30 (7.4%)	0.001
	No	54 (71.1%)	376 (92.6%)	
Are you on hormonal therapy?	Yes	10 (13.2%)	13 (3.2%)	0.001
	No	66 (86.8%)	393 (96.8%)	
For females, are you on contraceptive pills?	Yes	24 (31.6%)	111 (27.3%)	0.035
	No	43 (56.6%)	275 (67.7%)	
Are you in menopause?	Yes	2 (2.6%)	3 (0.7%)	0.261
	No	50 (65.8%)	288 (70.9%)	
Type of delivery	Vaginal	14 (18.4%)	77 (19.0%)	0.002
	C-section	13 (17.1%)	30 (7.4%)	
	Both	8 (10.5%)	16 (3.9%)	
How many children do you have?	None	32 (42.1%)	242 (59.6%)	0.004
	1 child	15 (19.7%)	47 (11.6%)	
	2 children	20 (26.3%)	52 (12.8%)	
	3 children	2 (2.6%)	38 (9.4%)	
	4 children	4 (5.3%)	18 (4.4%)	
	5 children	2 (2.6%)	5 (1.2%)	
	6 children	1 (1.3%)	4 (1.0%)	

Compared to nurses with varicose veins and without varicose veins according to the continuous variables, age was 31.5 ± 5.5 years versus 30.4 ± 5.5 years (p = 0.101), height was 158.3 ± 8.3 cm versus 159 ± 21.9 cm (p = 0.788), and weight was 62.8 ± 16.6 kg versus 62.1 ± 14.4 kg (p = 0.715). Moreover, the hours standing per shift was 7.8 ± 4.2 hours versus 7.5 ± 3.1 hours (p = 0.497), the number of gravida was 2.68 ± 1.5 versus 2.32 ± 1.4 (p = 0.286), the number of parities was 1.89 ± 0.76 versus 1.91 ± 0.96 (p = 0.926), and the number of abortions was 1.25 ± 0.45 versus 1.65 ± 1.18 (p = 0.253). All these variables did not exhibit a statistically significant correlation with varicose veins (p > 0.05). However, the hours of sitting per shift was 4.5 ± 3.8 hours versus 3.1 ± 3 hours (p = 0.002), indicating statistically significant with varicose veins (Table 3).

**Table 3:** Comparison of continuous variables between nurses with and without varicose veins.

Variable	With varicose veins mean (SD)	Without varicose veins mean (SD)	p-value
Age by years	31.5 (±5.5)	30.4 (±5.5)	0.101
Height by cm	158.3 (±8.3)	159 (±21.9)	0.788
Weight by kg	62.8 (±16.6)	62.1 (±14.4)	0.715
Hours standing per shift	7.8 (±4.2)	7.5 (±3.1)	0.497
Hours sitting per shift	4.5 (±3.8)	3.1 (±3)	0.002
Number of gravida	2.68 (±1.5)	2.32 (±1.4)	0.286
Number of parity	1.89 (±0.76)	1.91 (±0.96)	0.926
Number of abortions	1.25 (±0.45)	1.65 (±1.18)	0.253

## Discussion

In our study, the prevalence of varicose veins among nurses was 15.8%. The prevalence of varicose veins was higher in female nurses compared to male nurses. Varicose vein prevalence reported in previous research has differed widely, ranging from 2% to 56% in males and 1% to 73% in females [14,15]. A cross-sectional survey was conducted in 2020 in Saudi Arabia with 366 nurses, with 322 females (88.0%) and 44 males (12.0%). There were 40 (11%) patients with a confirmed diagnosis of varicose veins, of whom 39 were female and one male [16]. However, because our study included a large differential in the number of female and male nurses who participated, these findings cannot be inferred.

Varicose veins were common in nurses who worked at King Fahd Central Hospital, 53.9%, compared with 46.1% at Prince Muhammad bin Nasser Hospital. This could be attributed to the difference in workload, the number of staff, and the number of patients. Both hospitals are located in Jazan City, southwest of KSA. Among the study participants, 40.90% worked in the medical ward, 19.30% in the emergency department, 7.50% in the intensive care unit, 6.60% in the cardiac care unit, and 25.7% in other departments. Most nurses work in the medical ward because both hospitals receive many inpatients daily.

In our study, the risk factors for varicose veins were ethnicity, lifting heavy objects, lack of exercise, family history, use of hormonal therapy, use of contraceptive pills, type of delivery, and number of children. All these risk factors showed a statistically significant association with varicose veins. The varicose vein predictors that were not statistically significant in this study (p > 0.05) were gender, social status, place of work, number of shifts, hours standing per shift, years of work, and smoking. Age, sex, and ethnic origin were shown to be risk factors for varicose veins in most previous research [17]. In our study, the number of shifts and standing hours were not risk factors for varicose veins in King Fahd Central Hospital and Prince Muhammad bin Nasser Hospitals. There is a difference between our study and the previous studies, which could be attributed to the difference in workload, lifestyle, social life, and type of socks. Nurses in our study worked only eight hours per shift, some wore bandage socks while on the job, and 35.5% of nurses had only worked for one to four years. AlBader et al. found that based on shifts per week (p = 0.23) and standing hours (p = 0.09) [16].

Ethnicity was considered a risk factor for varicose veins in this study, as 88.2% of nurses who had varicose veins were Arabian, compared with 10.5% Asian and only 1.3% African. However, these findings were not valid and could not be inferred since the number of participating nurses significantly varied by ethnicity. Mekky et al. reported that varicose veins were investigated in 504 female cotton workers in England and 467 female cotton workers in Egypt. Varicose veins were far more prevalent in the English mill population than in the Egyptian community [18].

The nurses with a family history had more varicose vein incidents (64.5%) than the nurses who did not have a family history (35.5%). Cornu-Thenard et al. studied 143 families, including 67 patients and their parents and 67 controls and their parents. A total of 402 patients were tested. The findings indicated that inheritance had a significant impact on the development of varicose veins (p = 0.001). Children had a 90% chance of acquiring varicose veins if both parents had the illness, 25% for men and 62% for females if only one parent had it, and 20% for neither parent [19].

In this research, 68.4% of participants diagnosed with varicose veins usually lift objects that weigh 23 kg or more during work time and lift heavy objects such as electronic devices and equipment like kidney machines and oxygen tanks. Moreover, carrying the patient to transfer him from one ward to another ward increases pressure on the lower limbs and leads to vascular wall and valvular damage. The research by Tabatabaeifar et al. corroborated our study in that the prolonged lifting of large things was associated with varicose veins and a higher risk of surgical therapy [20]. In our study, 63.2% of nurses who did not play sports and had varicose veins, compared with 36.8% of nurses with varicose veins who did. Abou-ElWafa et al. reported that varicose veins were substantially more frequent among nurses with the following characteristics: over the age of 25, married, no sports, and obese [21]. The use of hormonal therapy included conjugated equine estrogens with or without medroxyprogesterone acetate. In addition, contraceptive pills containing estrogen and progesterone or progesterone alone increased the incidence of varicose veins [22,23].

The nurses who delivered vaginally had a higher chance of developing varicose veins than the nurses who delivered through C-sections. Additionally, the females with children had a greater risk than the females who did not. This is because when a female is pregnant, progesterone levels rise, and the uterine wall expands, increasing blood volume and potentially resulting in valve failure. Jawien et al. reported that varicose veins were more prevalent in women than in males. The number of pregnancies (more than two pregnancies) considerably separated women with and without varicose veins [24].

When we compared the nurses with varicose veins and those without varicose veins according to the continuous variables, we found that age (p = 0.101), height (p = 0.788), weight (p = 0.715), and hours standing per shift (p = 0.497) did not show statistically significant associations with varicose veins (p > 0.05). The number of hours sitting per shift was statistically significant (p = 0.002). Chen and Guo reported that in a comparison of hairdressers regarding varicose veins, those with lower limb varicose veins had longer work histories (30.5 vs. 24.0 years, p = 0.005), higher ages (49.3 vs. 44.7 years, p = 0.032), and worked more hours standing each month (213.9 vs. 176.0 hours, p = 0.008) [25]. Our study revealed no statistically significant association between varicose veins and the number of gravida (p = 0.286), the number of parity (p = 0.926), and the number of abortions (p = 0.253). In contrast, a previous study by Laurikka et al. found an increased incidence of varicose veins as the number of births increased [26]. Coughlin et al. found an association between multiparity and varicose veins in pregnant women [27].

In our study, the comorbidities associated with varicose veins were deep vein thrombosis (p = 0.001), hypertension (p = 0.002), chronic constipation (p = 0.006), diabetes (p = 0.001), kidney disease (p = 0.001), rheumatoid arthritis (p = 0.001), coronary artery disease, and severe occupational injury to the lower extremities (p = 0.001). This conclusion was consistent with the report by Bermudez et al. that insufficiency in deep veins was higher in traumatized limbs than in non-traumatized limbs [28]. Varicose veins are a sign of persistent venous hypertension or high blood pressure in the veins. High blood pressure in veins irreversibly destroys valves and weakens vein walls. This illness may cause swelling, soreness, heaviness, exhaustion, and other symptoms like leg ulcers and blood clots.

Nurses are responsible for most of the health system's services. Increasing varicose intensity can impair work output, threaten physical and mental health - particularly in older adults - and impose high costs on individuals and healthcare systems. Based on the results of this study, increasing the number of nursing workers for patient care, encouraging physical exercise, and bringing the pension age forward appear to be required to avoid the occurrence and severity of varicose veins.

Limitations

The study's limitations include the tiny sample size. The cross-sectional design precluded the investigation of variations in varicose veins over time. Additionally, we were unable to ascertain the participants' lifestyle and exercise habits. Furthermore, this research included nurses from two hospitals only; therefore, the findings may not be generalizable to other populations.

## Conclusions

This cross-sectional study contributes to the knowledge of the prevalence and risk factors for varicose veins. Our study has revealed a low prevalence of varicose veins among nurses working at King Fahd Central Hospital and Prince Muhammad bin Nasser Hospital. The prevalence of varicose veins was higher in females than in males. The varicose vein risk factors were ethnicity, lifting heavy objects, lack of exercise, family history, use of hormonal therapy, use of contraceptive pills, type of delivery, the number of children, and hours sitting per shift. The comorbidities associated with varicose veins were deep vein thrombosis, hypertension, chronic constipation, diabetes, kidney disease, rheumatoid arthritis, coronary artery disease, and severe occupational injury to the lower extremities. Nurses are responsible for most health system services, and hiring more nursing workers for patient care, promoting physical exercise, and lowering the pension age seems to be required to prevent the occurrence and development of varicose veins.
